# CLDN1 expression in cervical cancer cells is related to tumor invasion and metastasis

**DOI:** 10.18632/oncotarget.13871

**Published:** 2016-12-10

**Authors:** Wei-na Zhang, Wei Li, Xiao-li Wang, Zheng Hu, Da Zhu, Wen-cheng Ding, Dan Liu, Ke-zhen Li, Ding Ma, Hui Wang

**Affiliations:** ^1^ Cancer Biology Research Center, Tongji Hospital, Tongji Medical College, Huazhong University of Science and Technology, Wuhan, Hubei 430030, P.R. China; ^2^ Department of Gynecology, Qingdao Municipal Hospital, Qingdao, Shandong 266000, P.R. China

**Keywords:** cervical cancer, CLDN1, array CGH, EMT

## Abstract

Even though infection with human papillomaviruses (HPV) is very important, it is not the sole cause of cervical cancer. Because it is known that genetic variations that result from HPV infection are probably the most important causes of cervical cancer, we used human whole genome array comparative genomic hybridization to detect the copy number variations of genes in cervical squamous cell carcinoma. The results of the array were validated by PCR, FISH and immunohistochemistry. We find that the copy number and protein expression of claudin-1 (CLDN1) increase with the progression of cervical cancer. The strong positive staining of CLDN1 in the cervical lymph node metastasis group received a significantly higher score than the staining in the group with no lymph node metastasis of cervical cancer tissues. The overexpression of CLDN1 in SiHa cells can increase anti-apoptosis ability and promote invasive ability of these cells accompanied by a decrease in expression of the epithelial marker E-cadherin as well as an increase in the expression of the mesenchymal marker vimentin. CLDN1 induces the epithelial-mesenchymal transition (EMT) through its interaction with SNAI1. Furthermore, we demonstrate that CLDN1 overexpression has significant effects on the growth and metastasis of xenografted tumors in athymic mice. These data suggest that CLDN1 promotes invasion and metastasis in cervical cancer cells via the expression of EMT/invasion-related genes. Therefore, CLDN1 could be a potential therapeutic target for the treatment of cervical cancer.

## INTRODUCTION

With the introduction of population-wide screening programs, the worldwide incidence and mortality rate of cervical cancer have continued to decline. However, cervical cancer remains the third most commonly diagnosed malignancy and the fourth leading cause of cancer death in females worldwide [1]. Persistent infection with high-risk human papillomavirus (HPV) is recognized as a necessary initiating event but not a sufficient event in cervical carcinogenesis [2]. The integration of HPV into the human genome is recognized as an important basis for cervical carcinogenesis [3]. The high-risk forms of HPV can induce genomic instability, such as chromosome segregation, aneuploidy, and structural chromosome aberrations, in normal human cells, which is one of the hallmarks of a cancer cell. This activity is likely to be functionally correlated to the contribution of high-risk HPV to malignant progression [4].

Chromosomal aberrations are one of several mechanisms that can lead to gene dysregulation and have long been known to stimulatethe pathogenesis of human cancers. The identification of regions of genomic gains and losses has resulted in the discovery of novel oncogenes and tumor suppressor genes, respectively [5, 6, 7]. Nonetheless, no reliable chromosomal alteration pattern that clearly defines cervical cancer and its precursor lesions has been identified.

Array Comparative Genomic Hybridization (aCGH) is a technique whereby an alteration profile in terms of gain and loss of chromosomal regions is obtained [8, 9]. As the resolution of these arrays has improved over the years, array CGH has become a powerful tool. This technique permits the high-resolution detection of DNA copy number imbalances, as well as the ability to map such copy number alterations throughout the genome [10]. CGH microarray has also been used to detect the fragments or genes that have been gained or lost in cervical cancer tissues. The aim of the present study is to identify genes that show high-frequency copy number changes and expression level changes that contribute to the biology and natural history of cervical squamous carcinoma. Additionally, we attempt to find some functional genes whose changes are correlatedto the progression and possibly the development of precursor lesions of cervical cancer.

We find that the copy number and protein expression of claudin-1 (CLDN1) increase with the progression of cervical cancer. The overexpression of CLDN1 in SiHa cells can increase anti-apoptosis ability and promote invasive ability of these cells and is accompanied by a decrease in the epithelial marker E-cadherin as well as an increase in the mesenchymal marker vimentin. CLDN1 overexpression can stimulate the growth and metastasis of xenografted tumors in athymic nude mice.

## RESULTS

### Validation of genomic alterations in cervical squamous cell carcinoma tissues

The high resolution of the human whole genome CGH array (Nimblegen 2.1M WG Tiling CGH array) was used to detect the genomic alterations in 10 frozen primary cervical squamous cell carcinoma tissues. The results showed that copy number gains were most frequently observed by array CGH at 3q27.3-3q29 (60%), 11q12.3-11q13.1 (50%), 3q23 (40%), xq28 (40%), 3q24-3q26.32 (30%), 5p15.1-5p15.3 (30%), 9q32-9q33.2 (30%), and 8q24.21 (20%) (Table 1). Copy number losses were most frequently observed on 19p13.3 (100%), 11p15.4-11p15.5 (90%), 20q13.3-20q13.33 (90%), 3p23 (80%), 14q11.1 (70%), 1p36.32-1p36.33 (70%), 7p22-7p22.3 (70%), 12q24.33 (60%), 13q34 (60%), 16q24.2-16q24.3 (60%), 17q25-17q25.3 (60%), 18q23 (60%), 3p25.1 (60%), 4p16.2-4p16.3 (60%), 10q26.3 (50%), 22q13.33 (50%), 5p15.33 (50%), 5q35.2-5q35.3 (50%), 17p11.2 (40%), and 2q35-37.3 (40%) (Table 2).

**Table 1:** The fragments and genes gained in cervical cancer

Cytoposition	Start(Mbp)	End(Mbp)	Frequency (%)	Genes
3q27.3-3q29	188.56	194.75	60	FAM79B, MIRN28, MIRN944, UNQ846, VEZF1L1, ATP13A5, LEPREL1, RTP4, ATP13A4, RTP2, CCDC50, UTS2D, HRASLS, OSTN, FGF12, CLDN16, CLDN1, TP63, IL1RAP, POP2, BCL6, LPP, SST
11q12.3-11q13.1	62.55	63.01	50	MGC34821, LOC387601, SLC22A9
3q23	140.76	141.9	40	CLSTN2
Xq28	153.71	154.86	40	FUNDC2, BRCC3, VBP1, CLIC2, LOC401622, F8V1, TMLHE
3q24-3q26.32	148.58	178.39	30	TNFSF10, SI, CP, TERC
5p15.1-5p15.3	3.64	16.67	30	DAP, TRIO, POLS, MTRR, ANKH
9q32-9q33.2	116.94	122.38	30	SNORA70C, MIRN147, ASTN2, DIPAS, PAPPA, CDK5RAP2, TRIM32, DBC1, TLR4
8q24.21	128.49	129.03	20	PVT1

**Table 2:** The fragments and genes lost in cervical cancer

Cytoposition	Start(Mbp)	End(Mbp)	Frequency	Genes
19p13.3	0.23	1.79	100	GPX4, KISS1R, STK11, PALM, BSG, MADCAM1, KISS1R, MUM1, CDC34,
20q13.3-20q13.33	59.26	62.37	90	TNFRSF6B, BIRC7, PTK6, EEF1A2, OGFR, GATA5
11p15.4-11p15.5	0.2	3.21	90	CARS, TH, INS, IGF2, H19, MUC6, MUC2, MUC5AC, MUC5B
3p21.3	48.57	50.61	80	RASSF1, SEMA3F, PRKAR2A
1p36.32-1p36.33	0.74	3.7	70	TNFRSF14, TNFRSF18, TNFRSF4, TP73
14q11.1	18.18	18.26	70	LOC441666
7p22-7p22.3	0.28	2.25	70	MAD1L1, MAFK, GPER
3p25.1	12.8	13.65	60	RPL32, NUP210, HDAC11
4p16.2-4p16.3	0.038	4.19	60	FGFR3, RNF4
12q24.33	130.34	132.24	60	POLE, CHFR, MMP17, GOLGA3, EP400
13q34	113.76	113.91	60	RASA3
16q24.2-16q24.3	85.92	88.69	60	MVD, APRT, MC1R, CYBA, FANCA, CDT1, CBFA2T3
17q25-17q25.3	76.62	78.6	60	CD7, RAC3, HGS, FASN, STRA13, MAFG, ASPSCR1
18q23	74.93	76.1	60	NFATC1, CTDP1
5q35.2-5q35.3	175.53	180.62	50	F12, FGFR4, SQSTM1, PDLIM7, FLT4
5p15.33	0.11	1.51	50	TERT, SLC6A3
10q26.3	133.76	135.32	50	CYP2E1, ADAM8, UTF1
22q13.33	48.87	49.56	50	ACR, ARSA
2q35-37.3	238.54	242.61	40	PER2, PDCD1, HDAC4, BOK
17p11.2	18.86	18.93	40	GRAP

**Figure 1:**
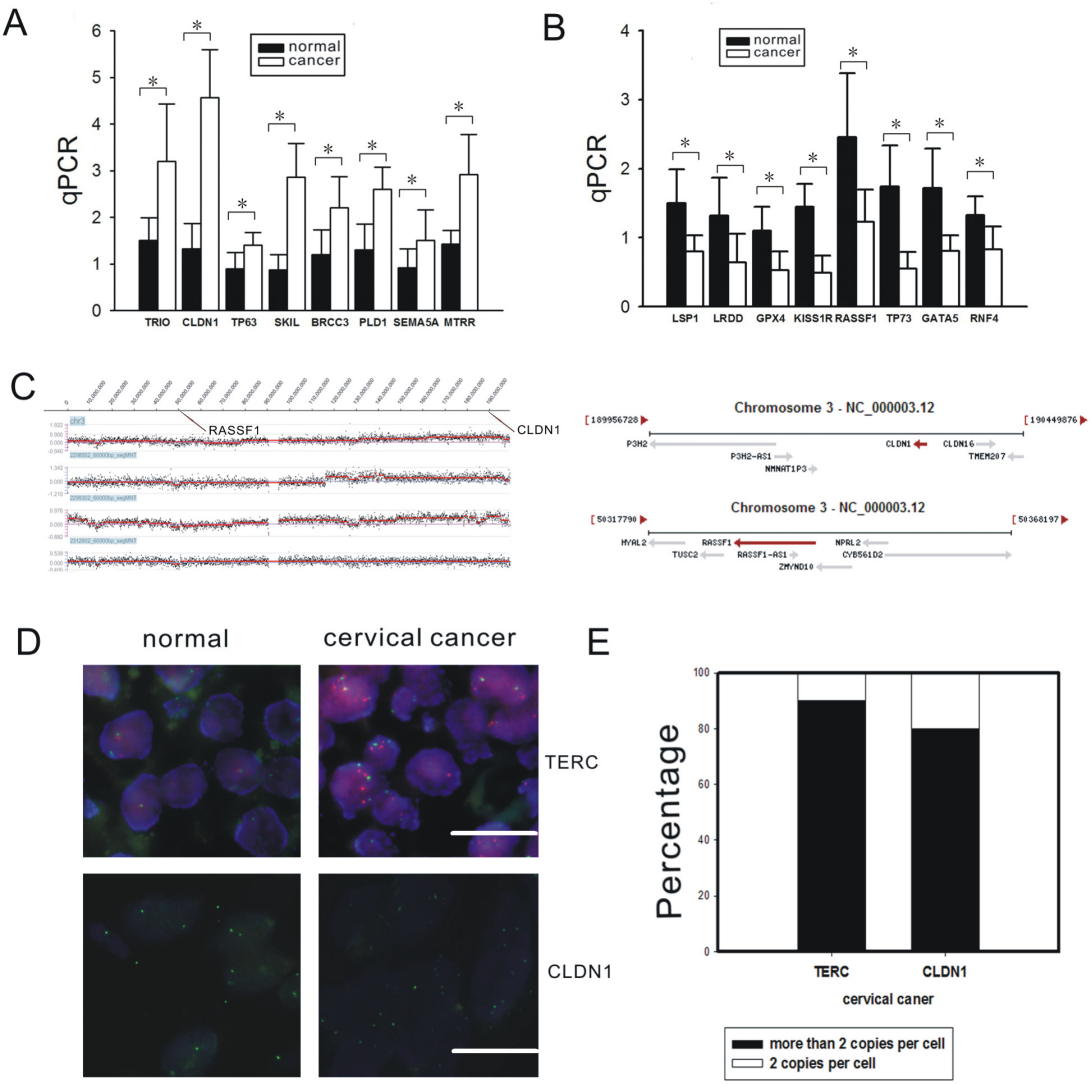
Validation of the array CGH results by qPCR and FISH A-B. qPCR was used to analyze eight interesting genes that were gained (A) and eight interesting genes that were lost (B) in 73 cervical cancer tissues and 20 normal cervical tissues. *: P<0.05. C. The dye intensities of chromosome 3 were analyzed by Signal Map software (Left). The genomic location of CLDN1 and RASSF1 were present (Right). D. The probes used were TERC (red), the centromere enumeration probe for chromosome 3 (green) and CLDN1 (green). (×400, bar: 20 μm). E. The percentages of cells with more than 2 copies of TERC and CLDN1 were counted. A minimum of 200 nuclei per case was scored.

To identify the correlations between gene expression levels and the gene copy numbers as seen on the array CGH, 16 genes that were derived from fragments with gain frequencies of more than 30% and with loss frequencies of more than 60% were selected for further validation by quantitative PCR (Figure 1A-1B). Significant correlations were observed in CLDN1 at 3q27.3-3q29 (P<0.01) (Location: 190305701-190322446) and RASSF1 at 3q23 (P<0.01) (Location: 50329786-50340936) (Figure 1A-1C).

Amplicons of TERC and CLDN1 were also tested by FISH using a BAC probe in the cervical squamous cell carcinoma tissue microarray. The copy numbers of TERC and CLDN1 in cancer tissues were both increased significantly compared with normal cervical tissue (Figure 1D-1E). The percentage of the counted cancer cells with 3-4 copies per cell of TERC was more than 90%, and the percentage of the counted cancer cells with 3-4 copies per cell of CLDN1 was more than 80%.

### Gained CLDN1 gene expression increases with the progression of cervical neoplasia

**Figure 2:**
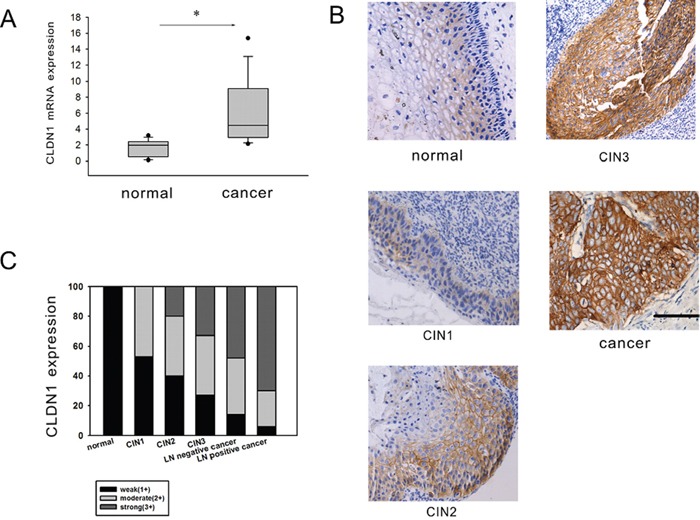
CLDN1 expression is increased in CIN and cervical cancer A. Reverse-transcription qPCR was used to detect the expression of CLDN1 mRNA in 73 fresh cervical cancer tissues and 20 normal cervical tissues. *: P<0.05. B. The expression of CLDN1 protein was examined by immunohistochemistry in the cervical cancer tissue microarrays, CIN tissue microarrays and paraffin-embedded sections of cervical cancer tissues and normal cervical tissue (×200, bar: 50 μm). C. The different grade of positive staining percentage of the CLDN1 staining in different cervical diseases.

Claudin proteins represent a large family of integral membrane proteins that are crucial for the formation and function of tight junctions. The expression of CLDN1 mRNA was firstly tested using reverse transcription PCR in 73 fresh cervical cancer tissues and 20 normal cervical tissues. The results showed that the expression of CLDN1 mRNA was increased significantly in cervical cancer tissues (p<0.05) (Figure 2A). To clarify the role of CLDN1 in the development of cervical lesions, the expression of CLDN1 protein was detected by immunohistochemistry in tissue microarray (CR805, CIN481) and in paraffin-embedded sections of 73 cervical cancer tissue samples and 20 normal cervical tissue samples. The expression of CLDN1 protein was significantly increased from normal cervical tissues to cervical cancer tissues (Figure 2B). According to an analysis of the CLDN1 immunostaining scores of cervical cancer tissue with or without lymph node metastasis (Supplementary Table 1), we found that the strong positive staining for CLDN1 in the cervical lymph node metastasis group was significantly higher than the group with no lymph node metastasis of cervical cancer tissues (p < 0.05) (Figure 2C). This result suggests that a high expression of CLDN1 may play an important role in cervical cancer metastasis. To clarify the role of CLDN1 in the development of cervical lesions, we tested the expression of CLDN1 in a cervical intraepithelial neoplasia (CIN) tissue microarray (Figure 2B). The results showed that strong positive expression of CLDN1 increases with the progression of CIN (i.e., from CIN1 to CIN3).

### CLDN1 is important for apoptosis, invasion and cell transformation of cervical cancer cells

**Figure 3:**
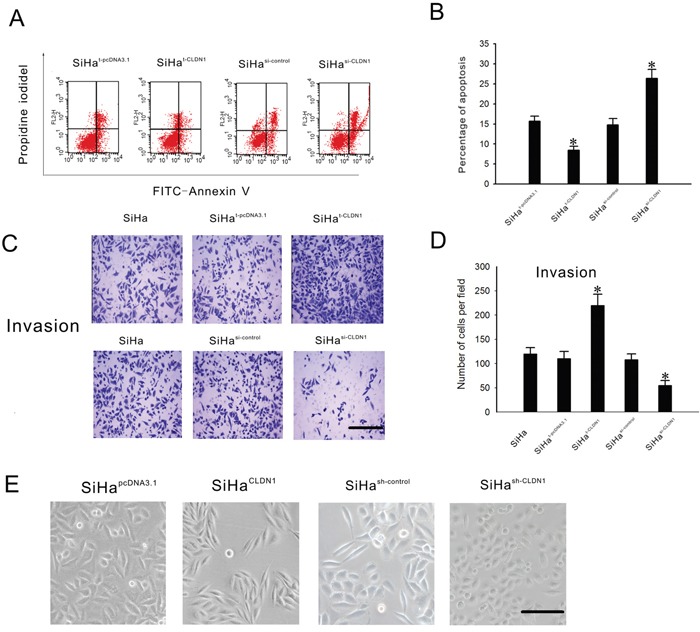
Effects of the inhibition of CLDN1 expression on apoptosis, invasion and cellular transformation in SiHa cells A. Taxol (1 mmol/L) was added to SiHat-pcDNA3.1 cells, SiHat-CLDN1 cells, SiHasi-control cells or SiHasi-CLDN1 cells, respectively. The cells were harvested after 48 h for Annexin V analysis. B. Representative FACS analyses and a summary of Annexin V and PI staining shown in (A). C. The cell invasion analysis was performed using a 24-well plate with Matrigel-coated Transwell inserts. In all, 105 SiHat-pcDNA3.1 cells, SiHat-CLDN1 cells, SiHasi-control cells or SiHasi-CLDN1 cells were cultured in the upper plate, respectively. And 200 μl of fetal bovine serum was placed in the lower plate. After 24 hours in culture, the cells that had migrated through the filter were counted(×100, bar: 100 μm). D. A summary of the cell invasion analysis shown in (C). Each bar represents the mean ± SD of 3 experiments. E. Representative phase-contrast images of the stable clones SiHapcDNA3.1, SiHaCLDN1, SiHash-control and SiHash-CLDN1 cells grown in monolayer cultures(×200, bar: 50 μm). * p<0.05, compared with the relative control.

To clarify whether CLDN1 protein expression has a causal role in tumor progression and invasion, we first tested the expression levels of CLDN1 in different cervical cancer cell lines. The results showed the expression level of CLDN1 protein was higher in SiHa cells than in other cells (data not shown). A CLDN1 expression plasmid and small interfering RNA (siRNA) targeting CLDN1 were transiently transfected into SiHa cells. SiHa cells were induced to undergo apoptosis by taxol. And cell apoptosis was quantified 48 h later by Annexin V and PI staining. As shown in Figure 3A-3B, the transient forced expression of CLDN1 resulted in a significant decrease in apoptosis in SiHa cells, whereas transient CLDN1 knockdown led to a significant increase in apoptosis in SiHa cells. The ability of CLDN1 to affect the proliferation and colony formation of cancer cells was also detected using a CCK8 assay and a colony formation assay, respectively (Supplementary Figure 2). The results did not reveal any significant difference between the three groups in terms of cell proliferation and colony formation. In addition, using a Boyden chamber invasion assay, we observed a significant increase in the invasive capacity of SiHa^t-CLDN1^ cells and a significant decrease in the invasive capacity of SiHa^si-CLDN1^ cells (Figure 3C-3D).

Stable CLDN1-overexpressing (SiHa^CLDN1^) and CLDN1 knockdown (SiHa^sh-CLDN1^) cell clones were selected. The SiHa^CLDN1^ cells appeared spindly and had a fibroblastoid appearance, while the SiHa^sh-CLDN1^ cells exhibited an epithelial morphology and were easily able to form aggregates in monolayer culture (Figure 3E). The changes in the appearance of SiHa^CLDN1^ cells resembled the process of the epithelial-mesenchymal transition (EMT), which may stimulate the invasive nature of cancer cells.

### CLDN1 induces functional changes in the markers of the epithelial-mesenchymal transition

**Figure 4:**
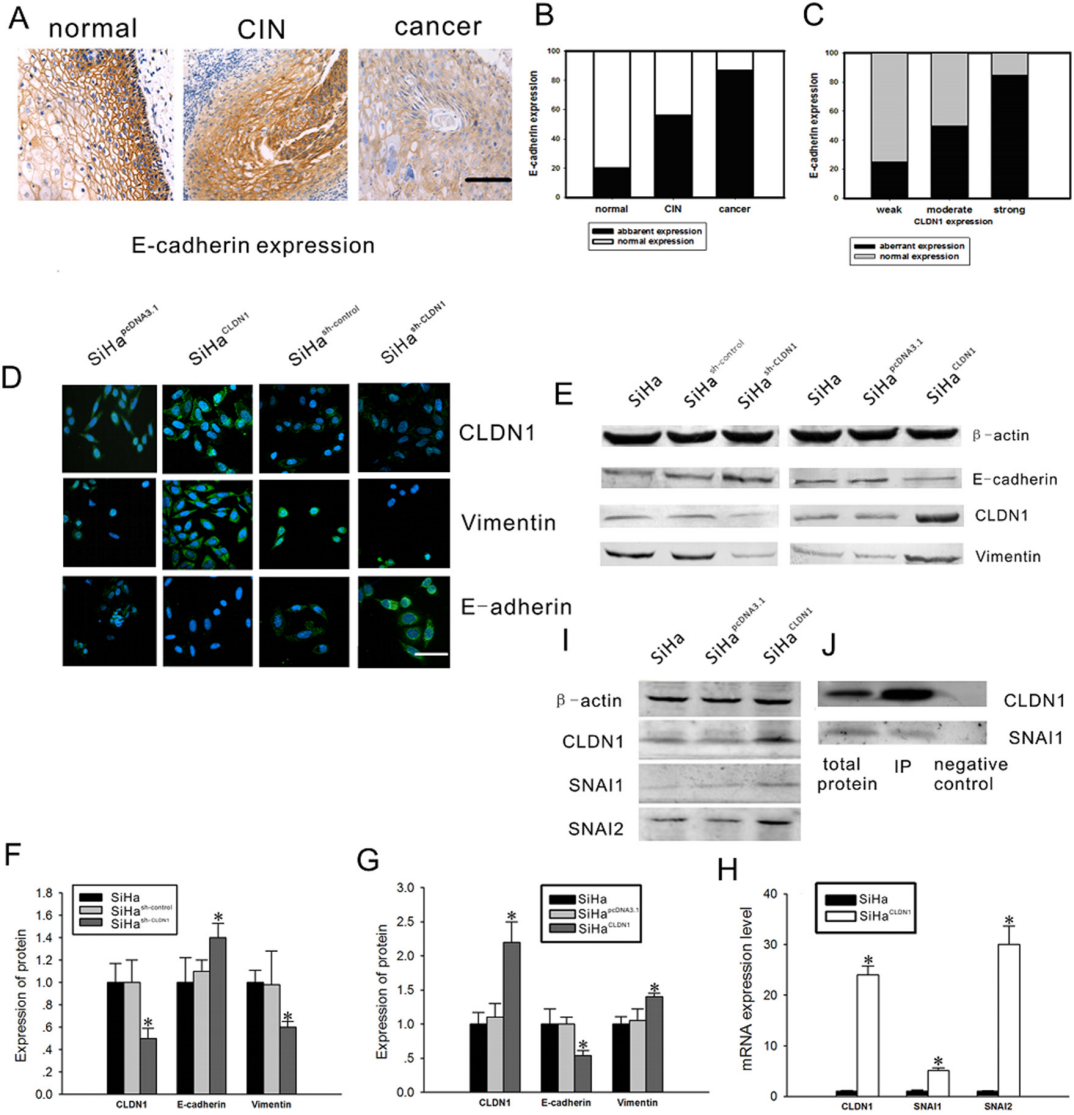
Effects of CLDN1 on epithelial-mesenchymal markers A. E-cadherin expression was detected by immunohistochemistry in 15 normal cervical tissues, 45 CIN tissues and 69 cervical cancer tissues (×200, bar: 50 μm). B. A summary of the analysis of aberrant E-cadherin expression shown in A. C. The relationship between aberrant E-cadherin expression and CLDN1 expression was analyzed. D. The immunofluorescence staining of CLDN1, vimentin and E-cadherin in SiHapcDNA3.1 cells, SiHaCLDN1 cells, SiHash-control cells and SiHash-CLDN1 cells(×200, bar: 25 μm). E. CLDN1, vimentin and E-cadherin protein expression was examined by western blot in SiHa cells, SiHapcDNA3.1 cells, SiHaCLDN1 cells, SiHash-control cells and SiHash-CLDN1 cells. The statistical results of the western blot analysis shown in F. and G. Each bar represents the mean ± SD of 3 experiments. H. The expression of SNAI1 and SNAI2 mRNA in SiHaCLDN1 cells was demonstrated by reverse transcription qPCR (RQ). Each bar represents the mean ± SD of 3 experiments. I. The expression of CLDN1, SNAI1, and SNAI2 protein in SiHaCLDN1 and SiHapcDNA3.1 cells. J. Coimmunoprecipitation of SNAI1 and CLDN1 from SiHa cells. Protein extracts from SiHa cells were immunoprecipitated with the indicated antibodies, electrophoresed on a 7% SDS-PAGE gel, and immunoblotted with an anti-SNAI1 antibody. * p<0.05, compared with the relative control.

EMT is accompanied by the loss of cell-cell contacts and the acquisition of migratory and motile properties that may contribute to cancer progression. The loss of E-cadherin expression seems to be heavily involved in EMT, and therefore, E-cadherin has emerged as one of the maintainers of the epithelial phenotype [11]. The normal expression of E-cadherin is characterized by strong or moderate membranous staining with weak or negative cytoplasmic staining. Other expression patterns have been regarded as aberrant immunoreactivity [12]. First, E-cadherin expression was detected in cervical cancer tissues and CIN tissues by immunohistochemistry (Figure 4A). With the progression of cervical neoplasia, the percentage of the cells with aberrant E-cadherin expression increased from 20% (3/15) in normal tissues and 56% (25/45) in CIN tissues to 87% (60/69) in cervical cancer tissues (Figure 4B). Moreover, the increased CLDN1 expression was correlated to the aberrant expression of E-cadherin (Figure 4C). The expression of vimentin and E-cadherin was also examined by immunofluorescence and western blot. Compared with the control cells, increased expression of vimentin and decreased expression of E-cadherin were observed in CLDN1-overexpressing cells (Figure 4D-4E, 4G), whereas decreased expression of vimentin and increased expression of E-cadherin were found in CLDN1-knockdown cells (Figure 4D-4F).

It was reported that the activation of transcriptional regulators such as snail (SNAI1) and slug (SNAI2) repress the expression of E-cadherin, which is related to the progression of EMT [13]. Hence, mRNA and protein expression of SNAI1 and SNAI2 were detected by PCR and western blot in CLDN1-overexpressing SiHa cells. It was demonstrated that the expression of both SNAI1 and SNAI2 increased in CLDN1-overexpressing cells (Figure 4H-4I). Furthermore, immunoprecipitation was used to detect the relationships between CLDN1 and transcription factors in SiHa cells (Figure 4J). It was shown that CLDN1 had a direct or an indirect bind with SNAI1. Nonetheless, a similar result was not found for SNAI2.

### Assessment of tumor growth and metastatic potential according to CLDN1 expression *in vivo*

**Figure 5:**
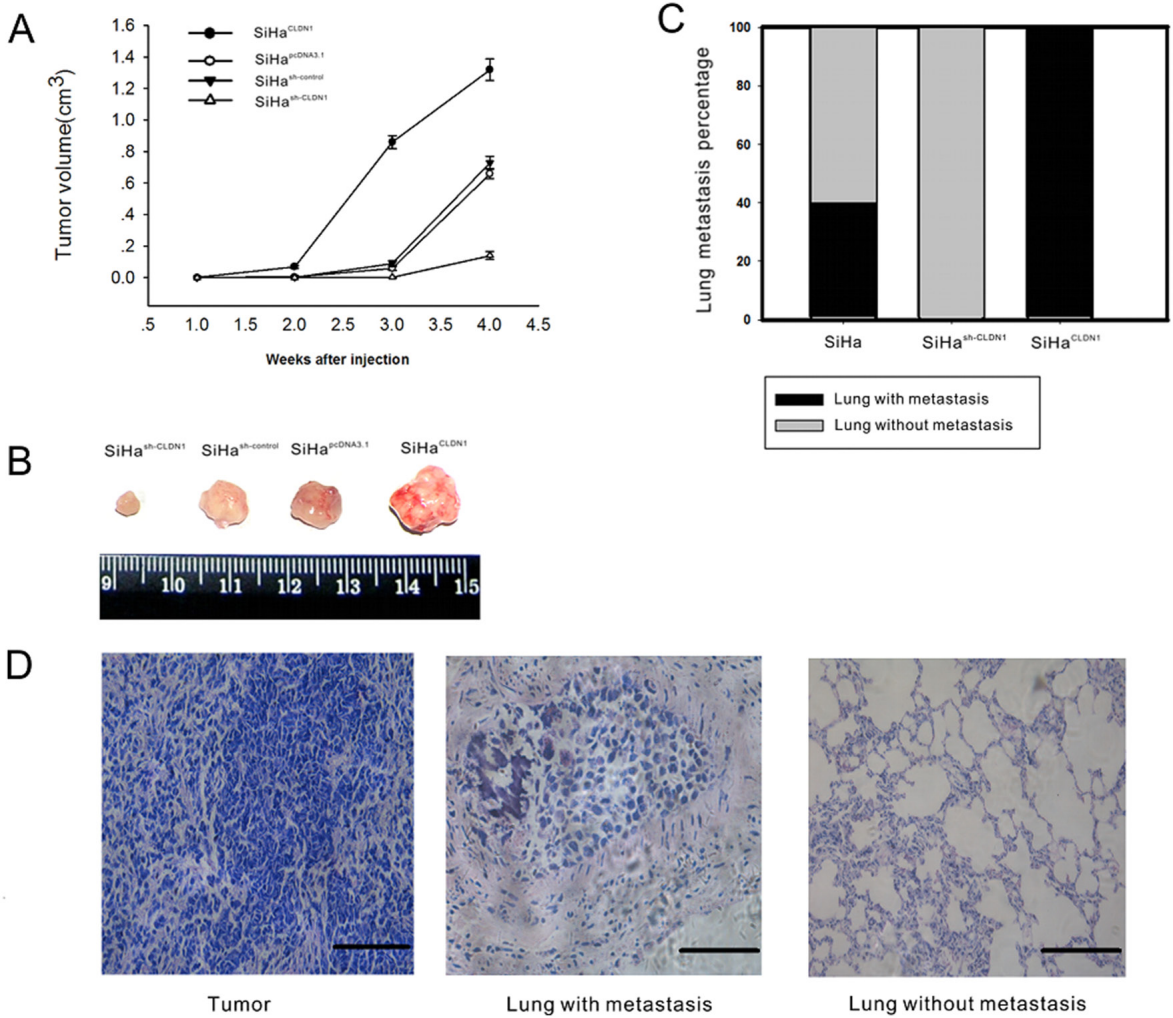
Overexpression CLDN1 prompts tumor growth and lung metastasis *in vivo* A. Tumorigenicity was monitored after the subcutaneous inoculation of 5 × 107 cells (SiHapcDNA3.1, SiHaCLDN1, SiHash-control or SiHash-CLDN1) into the flanks of 4-week-old female athymic nude mice. The data are shown as the mean ± SD of the 5 mice in each group. B. The subcutaneous tumors were removed 4 weeks after inoculation in the groups described in (A). C-D. Lung metastasis. In all, 5 × 106 SiHa cells, SiHaCLDN1 cells, or SiHash-CLDN1 cells were injected into the tail vein of female athymic nude mice (5 weeks old; n = 5), respectively. The percentage of mice with lung metastasis after 8 weeks in the three groups is shown (C). (D) H&E staining of subcutaneous tumors (×200, bar: 50 μm), lungs with tumor metastasis (×400, bar: 25 μm), and lungs without tumor metastasis (×200, bar: 50 μm).

To study the effect of CLDN1 on tumor growth, stable clones of SiHa^pcDNA3.1^, SiHa^CLDN1^, SiHa^sh-control^ and SiHa^sh-CLDN1^ were injected subcutaneously into the flanks of nude mice. The larger subcutaneous tumors appeared in mice that were injected with SiHa^CLDN1^ cells, whereas smaller subcutaneous tumors were found in mice that were injected with SiHa^sh-CLDN1^ cells (Figure 5A-5B). Furthermore, to determine the effect of CLDN1 on metastatic potential, SiHa, SiHa^CLDN1^ and SiHa^sh-CLDN1^ cells were injected into the tail veins of female nude mice. After 2 months, lung samples were analyzed for signs of metastasis. In the CLDN1 overexpression group, metastatic cancer cells were found in the lungs of mice (5/5), but in the control group, the metastatic rate was 2/5. No metastatic lesions were apparent in the CLDN1 knockdown group (Figure 5C-5D).

## DISCUSSION

This study demonstrated the presence of chromosomal aberrations such as genomic gains and losses in cervical cancer tissues by array CGH. Many increased copy number fragments were found in 3q, such as 3q27.3-3q29, 3q23, and 3q24-3q26.32. Additionally, many copy number losses were observed, including high frequency changes such as 19p13.3 (100%), 11p15.4-11p15.5 (90%), and 20q13.3-20q13.33 (90%). Some interesting genes were validated by PCR, and the changes in expression were consistent with what was detected by array CGH. Two genes with an increased copy number, TERC (3q24-3q26.32) and CLDN1 (3q27.3-3q29), were further detected by FISH. TERC gain has been found in SiHa cells and correlated to cervical cancer progression, which is consistent with our data [14, 15]. The number of copies of CLDN1 in cancer tissues was increased significantly compared with normal cervical tissues.

A gain of 3q is characteristic of cervical cancer and has been implicated as a key event in the progression of severe dysplasia to invasive cancer [16], but no definitive exploration of 3q with regard to its function was performed. CLDN1 is a member of the claudin family, which is a family of ∼17–27 kDa integral membrane tight junction (TJ) proteins that determine the size of the molecules that pass through the paracellular space in epithelial and endothelial tissues. There are few data available allowing an understanding of the molecular interactions between the claudins. CLDN1 has the interaction with CLDN3 but not CLDN2. The experiment in HeLa cells showed that CLDN1 and CLDN5 were heterotypically interacting with CLDN3 but not CLDN4 [17]. Expression of CLDN1 has been examined in a number of cancer types [18]. Both an increase and a decrease in CLDN1 protein expression have been shown to be associated with tumorigenesis. Loss of CLDN1 has been reported to stimulate the tumor progression and invasion in some cancers, including prostate cancer, breast cancer and melanocytic neoplasia, while in esophageal squamous cell carcinoma, increased expression of CLDN1 was correlated with tumor progression [19–22]. Several studies exploring CLDN1 expression in patient material from cervical cancer has been reported recently, which were consistent with our data [23–24]. According to our data, the expression of CLDN1 increased with the progression of cervical neoplasia and was correlated to lymph node metastasis. Further studies showed that CLDN1 could increase the invasive ability of SiHa cells, as well as their resistance to apoptosis. It was reported that claudin-1 expression had no significant effects on the invasive ability of Hela cells [25]. Nonetheless, our previous study showed that CLDN1 expression in Siha cells derived from squmous carcinoma of cervix was significantly higher than that in Hela cells derived from adenocarcinoma of cervix(data not shown). It was seemed that CLDN1 may play an important role in the invasion of squmous carcinoma of cervix by its high expression. Moreover, CLDN1 could promote an increase in the expression of the mesenchymal protein marker vimentin and a decrease in the expression of the epithelial protein marker E-cadherin. This molecular change was related to the cellular transformation induced by CLDN1 overexpression.

EMT is accompanied by the loss of cell-cell contacts as well as by the acquisition of migratory and motile properties. Hence, EMT can have adverse effects on the organism and may contribute to pathological processes such as fibrosis and cancer [26]. EMT allows for the invasion and intravasation of tumor cells into the surrounding tissues, blood and lymphatic circulation [11, 12]. It was reported that EMT is correlated to the invasive ability of cervical carcinoma and the progression of malignant tumors [27, 28, 29]. In the mesenchymal trans-differentiation process of epithelial cells, the down-regulation of E-cadherin has been characterized as the major marker that is responsible for the loss of cell-cell contacts in EMT events [29]. E-cadherin is known to be regulated at the transcriptional level by the zinc finger transcription factors Snail and Slug. Both of these transcription factors inhibitthe expression of E-cadherin [30, 31, 32]. Nonetheless, studies on the roles of EMT and the underlying regulatory signals in cervical carcinogenesis are limited. In our study, it was demonstrated that the overexpression of CLDN1 could stimulate the expression of SNAI1 and SNAI2 in SiHa cells. CLDN1 had a direct or an indirect interaction with SNAI1 in SiHa cells, which was demonstrated by immunoprecipitation; this result suggested that the consequence of overexpression of CLDN1 could increase the invasion of cervical cancer cells. Moreover, we found that the overexpression of CLDN1 could stimulate subcutaneous tumor growth and metastasis in athymic nude mice.

In conclusion, the copy number of CLDN1 was increased in cervical cancer, which might enhance cervical cancer cell anti-apotptosis ability and stimulate invasion and metastasis, mainly through EMT/invasion-related genes. Moreover, CLDN1 may be a potential prognostic marker and a candidate cell surface therapeutic target in cervical cancer.

## MATERIALS AND METHODS

This study was reviewed and approved by the Ethics Committee of Tongji Hospital, Tongji Medical College, Huazhong University of Science and Technology (HUST). All experimental protocols were approved by the Institutional Animal Care and Use Committee of HUST, and the study was performed in strict accordance with the ARRIVE (Animal Research: Reporting of *In Vivo* Experiments) guidelines.

### Cervical cancer cell lines, tissue samples and mice

Cervical cancer cell lines SiHa were obtained from the American Type Culture Collection (ATCC) (Manassas, VA, USA) and were cultured as recommended by the ATCC. Cervical cancer tissue microarray CR805 (50 cervical cancer tissues from clinical phase I to III and 10 normal cervical tissues) and cervical intraepithelial neoplasia (CIN) tissue microarray CIN481 (15 CIN I, 15 CIN II, 15 CIN III) were both obtained from Alenabio Company (Alenabio, Xian, China). Ten fresh cervical cancer tissues and matched ten blood samples from the same cervical cancer patients used for aCGH were gained from Tongji Hospital of Huazhong University of Science and Technology, which did not receive radiotherapy or chemotherapy before surgery. The pathological diagnosis of these patients was cervical squamous cell carcinoma and all the clinical phase were I. 73 cervical cancer tissues and 20 normal cervical tissues used for quantitative real time polymerase chain reaction (qPCR) and immunohistochemistry were also gained from Tongji Hospital of Huazhong University of Science and Technology, which were from clinical phase I to III. 4-6 weeks old female nude mice were maintained and housed at the animal facilities of Tongji Medical College, Huazhong University of Science and Technology, Wuhan, China.

### Array CGH profiling

Laser capture microdissection (LCM) was used to obtain the purest population of cancer cells from the tissues (Supplementary Figure 1A). The genomic DNA of the selected cancer tissues was extracted using a DNA extraction kit (QIAamp DNA Micro Kit 56304, QIAGEN, Valencia, CA, USA). Then, the extracted DNA was amplified using an amplification kit (QIAGEN, EPLI-g UltraFast Mini Kit, Valencia, CA, USA). Whole-blood genomic DNA from the same patient was extracted using a Gentra Puregene Blood Kit (QIAGEN, Valencia, CA, USA) (Supplementary Figure 1A). Genome-wide copy number alterations were analyzed by array comparative genomic hybridization (CGH) using a Nimblegen 2.1M WG Tiling CGH array. The tested genomic DNA and control blood DNA were labeled with random primers marked by Cy3 & Cy5, respectively. Labeled DNA was hybridized overnight with the array. The dye intensities were calculated, and the analysis was performed by Signal Map software.

### Fluorescence in situ hybridization (FISH)

The probe for the copy number test of TERC (GP medical, Beijing, China) consisted of a BAC clone that contains the human telomerase gene (*TERC*, labeled with rhodamine) and a centromere enumeration probe for chromosome 3 (CSP3, labeled with FITC). The clone used for the copy number test of CLDN1 was the BAC clone RP11-54L9, which was purchased from Invitrogen. Nick translation was used to label the CLDN1 probe with FITC. The tissue microarray (CR805, Alenabio, Xian, China) contained cervical cancer tissues and normal cervical tissues. The tissue microarray was heated at 65°C for 4 hours. Then, the microarray was incubated for 30 min in xylene to allow the removal of the coverslips. Next, the slides were incubated in a graded ethanol series of 100%, 95%, and 75% for 5 mins each. The slides were incubated for 40 min with a 60% acetic acid solution on a hot plate at 45°C. Then, the tissues on the array were digested in proteinase K for 20 min. After washes with 2× SSC solution, the slides were denatured for 10 min. Then, the probe was hybridized to the tissue microarray at 37°C overnight. Post-hybridization washes consisted of three washes in 50% formamide solution, one wash in 2× SSC, and one wash in 2× SSC 0.1% NP-40 for 5 min each at 45°C. A minimum of 200 nuclei per case was scored. The number of hybridization signals for the TERC and CLDN1 loci were counted as follows: 1, 2, 3, 4, or more than 4 hybridization signals. The percentages of cells with >2 copies of TERC and CLDN1 per nuclei were counted.

### Isolation of RNA and DNA and quantitative real-time PCR (qPCR)

Genomic DNA and total RNA were extracted from fresh cervical cancer tissues and normal cervical tissues using the QIAGEN genomic DNA extraction kit and RNeasy kit, respectively. The reverse-transcription step was performed as described in the manufacturer's instructions (QIAGEN, Valencia, CA). qPCR was used to detect the changes in copy number and in mRNA expression in the cancer tissues and normal tissues. The protocol for qPCR was performed according to the manufacturer's instructions (Fermentas, Burlington, ON, Canada). The following primers used were used, from 5’ to 3’: MTRR (TCCTTAAGCATGGGATCTTAACTCA - ATCATGTACATCCTTGGCCATATTC), RNF4 (CCC ATCTGCATGGACGGATAC-GAATCACGGAGGCACT GGCTA), SEMA5A (ACCCAAGCACTTAGTTTCAC CACTG - TTACATCAAGCGGCATAGCACAA), TRIO (TCAAACTGGCTGACTTTGGAGATG - GACAGGGTT CCCGAGGATGA), CLDN1 (GCATGAAGTGTATGAAG TGCTTGGA-CGATTCTATTGCCATACCATGCTG), BR CC3 (CATCTCCATTGAGGGCCAGAA - CACAGGA TCTTGGGCAGCTCTAC), KISS1R (GTGCAAGTTC GTCAACTACATCCAG - TCCACACTCATGGCGGT CA), GATA5 (AATGCCTGCGGCCTCTACA-ATGGT CTTTGGCTTCCGCTTC), RASSF1 (ACTTGCGGAA GCTGTTGGA - CGCTGCAGGATACGTAGGAA), LSP1 (AGAAATGTCAGCAGCCCAGGA - TCAATCTTGG AGATGGGCAAGTC), TP63 (AGCAGCAAGTTTCG GACAGTACAA-GGATGTCATCTGGATACCATGTG TG), TP73 (CCCACCACTTTGAGGTCACTTTC - GT CTTGGCGATCTGGCAGTAGAG), PLD1 (CCTGGA CATACTCTCATTTGGGTTG - GATCCTGCCCTTGA TGTGACACTA), SKIL (GGCATTGCTATCTTCATGT GAACC-GAAACATGGTCACCTTCCTGCTTT), GPX4 (GCCTTCCCGTGTAACCAGTT - GGAGTTCCACTTG ATGGCATT), and LRDD (CTCTCCTTGGCACCCT TGAAT - GGAGAAGAGCATGTGACGGAT). The PCR program was as follows: 95°C for 1 min, followed by 40 cycles of 95°C for 15 s, 60°C for 15 s and 72°C for 30 s. The melt curve program was 95°C for 15 s, 60°C for 1 min and 95°C for 30 s.

### Immunohistochemistry

The tissue microarrays (CR805, CIN481, Alenabio, Xian, China) and the paraffin-embedded cervical tumors and normal tissue samples obtained from our hospital were used for the detection of CLDN1 expression. The antibody for the immunohistochemistry experiments was purchased from Invitrogen. Immunohistochemistry was performed according to the manufacturer's instructions (Zhongshan Goldenbridge, Beijing, China). The immunohistochemical analyses were performed as previously described [33]. The negative control slides were processed similarly but with the omission of the primary antibody. The slides were scored as follows: +, weak staining; ++, moderate staining; +++, strong staining.

### Immunofluorescence

Coverslips were placed in a 12-well plate, and the cells were then seeded into the wells. After one day of culture, the cover glass was removed for immunofluorescence analysis. The cells were fixed in 75% ethanol for 30 min, permeabilized in 3% Triton X-100 for 5 min, and blocked in 5% normal goat serum in PBS at 37°C for 1 h. The cells were then incubated with rabbit anti-CLDN1 (1:50), rabbit anti-E-cadherin(1:50) and rabbit anti-vimentin (1:50) overnight at 4°C. After washing, the cells were incubated with goat anti-rabbit immunoglobulin G (IgG)-FITC (Jackson ImmunoResearch Laboratories, West Grove, USA.) for 30 min at room temperature. The slides were observed by fluorescence microscopy using a Leica DM4000B microscope (40x lens objective and 0.75 numerical apertures) with a Qimaging Retiga 1300 camera.

### Cell culture and transfection

Human cervical cancer cell lines were cultured in DMEM supplemented with 10% FBS. One day before transfection, the cells were seeded in 6-well cell culture plates at a final density of 60–70% confluence. Cells were transfected using Effectene Transfection Reagent (QIAGEN) according to the manufacturer's instructions.

The CLDN1 expression plasmid was constructed from plasmid pcDNA3.1 (+) using double digestion. The selected restriction enzyme sites were Hind III and BamHI. The restriction enzymes Hind III and BamHI were purchased from Takara (Japan). The primer sequences for the complete CLDN1 sequence were as follows: Sense: 5′ GTCAAGCTTGCCCACCTGCAAACTCT 3′; Antisense: 5′GCGGATCCTTTCAACATGATTTTCTCCT 3′. The small interfering RNA and pRNAT-U6.1/Neo-shRNA targeting CLDN1 were purchased from RIBO BIO Company. The effective siRNA sequence were as follows: (1) sense 5’ GCAAUAGAAUCGUUCAAGA dTdT 3’, antisense 3’ dTdT CGUUAUCUUAGCAAGUUCU 5’, which targeted GCAATAGAATCGTTCAAGA; and (2) sense 5’ GUAUGAAGUGCUUGGAAGA dTdT 3’, antisense 3’ dTdT CAUACUUCACGAACCUUCU 5’, which targeted GTATGAAGTGCTTGGAAGA. The sequences for the construction of the shRNA plasmid were as follows: shRNA-CLDN1–F: 5’ GATCCCGCAATAGAATCGTTCAAGATTCAAGAGATCTTGAACGATTCTATTGCTTTTTTCCAAA 3’, shRNA-CLDN1–R: 5’ AGCTTTTGGAAAAAAGCAATAGAATCGTTCAAGATCTCTTGAATCTTGAACGATTCTATTGCGG 3’.

SiHa cells were transiently transfected with pcDNA3.1-CLDN1 (SiHa^t-pcDNA3.1^) or small interfering RNA, which targeted CLDN1 (SiHa^si-CLDN1^). The transfection was performed with Lipofectamine 2000 (Invitrogen) according to the manufacturer's instructions. Four to six hours after transient transfection, the cells were cultured in fresh complete medium for 48 h before further experimentation.

SiHa cells were stably transfected with pcDNA3.1 or pcDNA3.1 CLDN1 and control shRNA or CLDN1 shRNA with Lipofectamine 2000. At 24 h after the initial transfection, SiHa cells were trypsinized and seeded in the corresponding medium supplemented with 600 μg/ml G418. SiHa^pcDNA3.1^, SiHa^CLDN1^, SiHa^sh-control^ and SiHa^sh-CLDN1^ cell clones were selected, amplified and then verified by reverse transcription PCR and western blot.

### Annexin V Staining and FACS Analysis

SiHa cells were trypsinized, washed twice with ice-cold PBS (pH 7.4), and resuspended in 1× binding buffer (10 mM HEPES, pH 7.4, 140 mM NaCl, 2.5 mM CaCl2) at a concentration of 1 ×10^6^ cells/ml. 100 μl of cell suspension was transferred to 5-ml plastic tubes; then, 5 μl of Annexin V-fluorescein isothiocyanate (PharMingen, San Diego, USA) and 4 μl of 0.5 mg/ml PI were added. The cells were gently vortexed and incubated in the dark at room temperature for 20 min. Four hundred microliters of binding buffer was added to each tube, and Annexin V staining was analyzed by flow cytometry within 1 h. Cells that were negative for both PI and Annexin V staining were determined to be live cells, while Annexin V-positive cells were determined to be early apoptotic cells. PI-positive and Annexin V-positive cells were determined to be cells that were primarily in the late stages of apoptosis. These experiments were repeated two or three times.

### Transwell invasion assay

After transfection with a CLDN1-expressing plasmid or CLDN1 siRNA, 200 μl of SiHa cells at a concentration of 5 × 10^5^ cells/ml were placed on the Transwell plates coated with Matrigel. A total of 200 μl of 3T3 cell supernatant was mixed with 200 μl of fetal bovine serum and was then added to the 24-well plate underneath the Transwell inserts. Then, the cells in the 24-well plate with the Transwell inserts were cultured at 37°C for 24 hours. The fluid in the Transwell insert was removed and treated with paraformaldehyde for 10 min. After washing with PBS, the Transwell insert was immersed in crystal violet for 5 min. The cells in the different groups were then counted underneath a microscope.

### Immunoprecipitation

SiHa cells were cultured in a cell culture plate. After washes with PBS, the plated cells were lysed in RIPA lysis buffer. In all, 100-500 μg total cellular protein was transferred to a 1.5-ml microcentrifuge tube. Then, the CLDN1 primary antibody or IgG(control) (Invitrogen, NY, USA) was added, and the cells were incubated with the antibody for 1 hour at 4°C. Protein A/G PLUS-Agarose (Santa Cruz, CA, USA) was added to the mixture, which was incubated at 4°C on a rocker platform overnight. After washes with PBS, the supernatant was discarded, and the pellet was resuspended in 40 μl of 1x electrophoresis sample buffer. Then, the samples were boiled for 2-3 min and were analyzed in 20-μl aliquots by SDS-PAGE.

### Western blot

A total of 30 mg of protein was loaded and subjected to sodium dodecyl sulfate-polyacrylamide gel electrophoresis (SDS-PAGE) followed by the transfer of the proteins to a polyvinylidene fluoride membrane. The membranes were then blocked with 10% non-immune serum for 1 h. The membrane was incubated with a primary antibody against CLDN1 (Invitrogen, NY, USA), E-cadherin (Abcam, Cambridge, Britain), vimentin (Abcam, Cambridge, Britain) or β-actin at 4°C overnight, then washed three times with PBS and 0.1% Tween-20. After the membrane was incubated with the secondary antibody at room temperature for 1 h, the labeling was detected by BCIP/NBT.

### *In vivo* studies

To study the effect of CLDN1 on tumor growth, the stable clones SiHa^pcDNA3.1^, SiHa^CLDN1^, SiHa^sh-control^ and SiHa^sh-CLDN1^ were injected subcutaneously into the flanks of nude mice. Tumorigenicity was assessed by subcutaneous inoculation of 5 × 10^7^ cells into the flank of 4-week-old female athymic nude mice, and the animals were assessed 4 weeks after inoculation. Five mice were used for each clone.

To assess the impact of CLDN1 overexpression or inhibition on metastasis *in vivo*, 5 × 10^6^ SiHa^CLDN1^, SiHa^sh-CLDN1^ or SiHa cells were injected into the tail vein of female athymic nude mice (5 weeks old; n = 5). Nude mice were sacrificed at 8 weeks, and the number and size of the metastatic tumor foci in the lungs were documented.

### Statistical analysis

Student's t-test and 2-sided test of proportions were used to determine statistical significance. One-way ANOVA was used for an analysis of more than two groups. P <0.05(*) was considered significant.

## SUPPLEMENTARY FIGURES AND TABLE



## Abbreviations

HPV: human papillomavirus
aCGH: Array Comparative Genomic Hybridization
CLDN1: claudin-1
EMT: epithelial-mesenchymal transition
CIN: cervical intraepithelial neoplasia.
