# Supplementary material for: CLDN1 expression in cervical cancer cells is related to tumor invasion and metastasis
Source: Oncotarget. 2016 Dec 10;7(52):87449–61. doi: 10.18632/oncotarget.13871 (PMC5350000; doi:10.18632/oncotarget.13871)
Supplement: Supplementary file 1 [file oncotarget-07-87449-s001.pdf]

CLDN1 expression in cervical cancer cells is related to tumor invasion and metastasis

SUPPLEMENTARY FIGURES AND TABLE

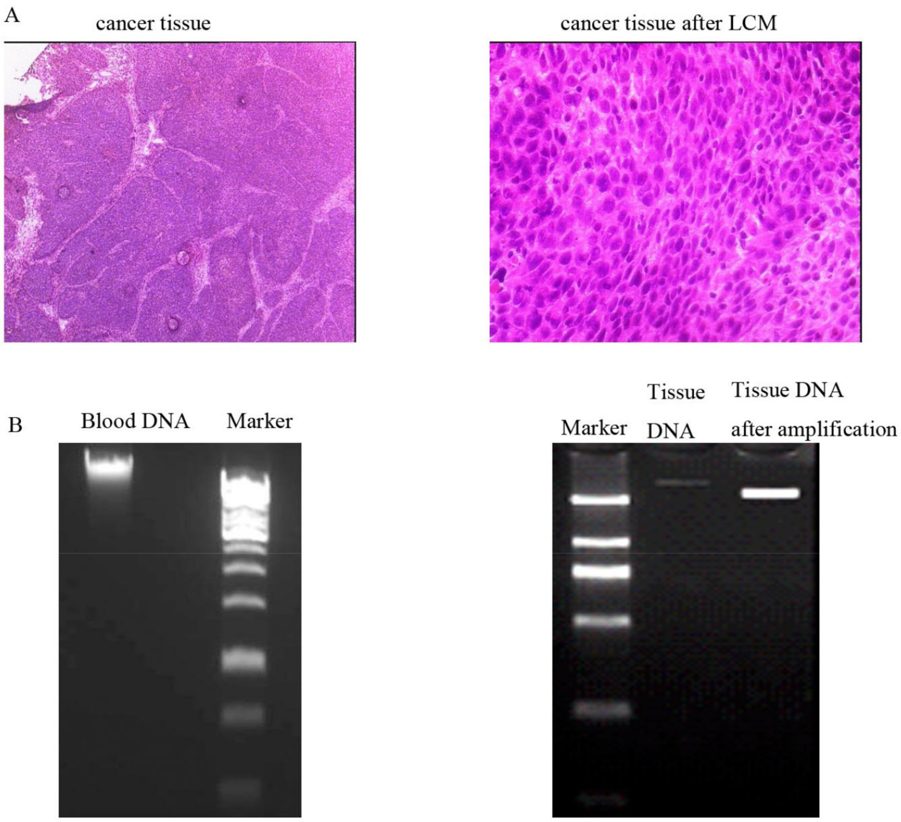

Supplementary Figure S1: The cancer tissues were selected by LCM and the extracted DNA was analyzed by array CGH. A. The cervical cancer tissues were stained by HE and the pure cancer tissues were selected by LCM. **B.** The whole blood genomic DNA of selected patient was extracted and tested by agar gel electrophoresis. The genomic DNA of the selected cancer tissues was extracted by DNA extraction kit and amplified by DNA amplification kit. Then the DNA was detected by agar gel electrophoresis.

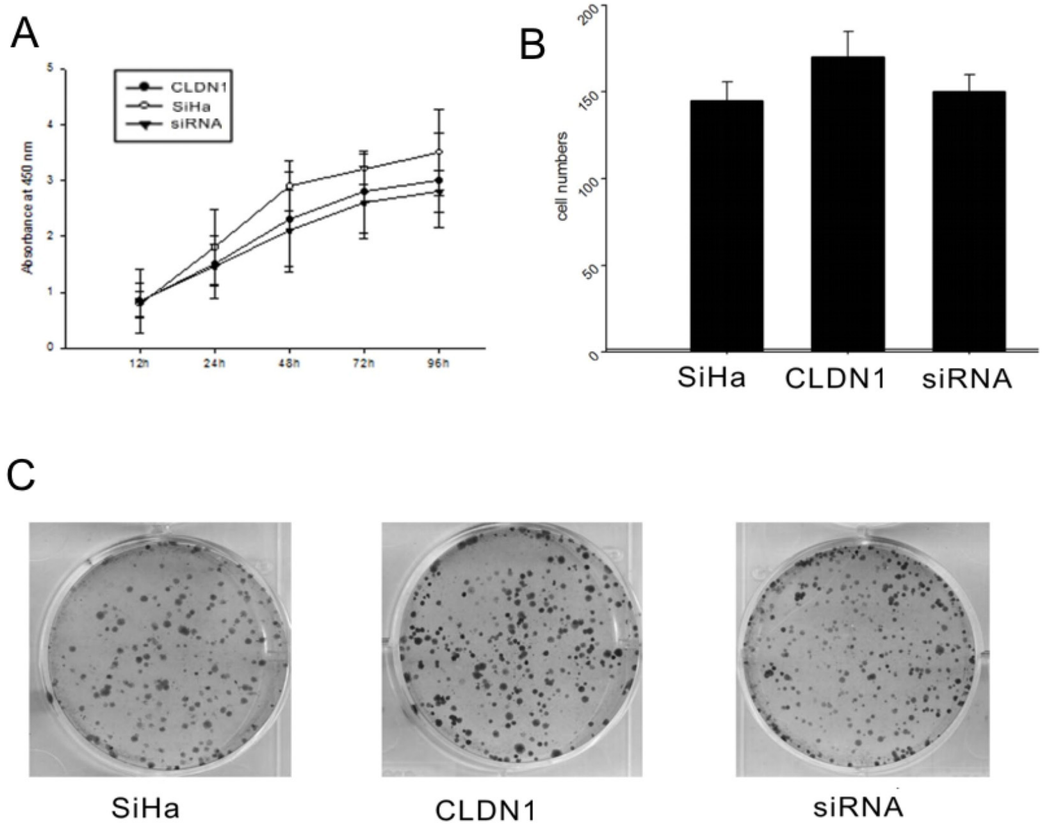

Supplementary Figure S2: The function of CLDN1 in SiHa cells. A. The proliferation of SiHa cells with different CLDN1 expression level was tested by cell counting kit-8(CCK8). 10000 SiHa cells/100ul with different CLDN1 expression level were planted in 96-well plate. CCK-8 solution was added to the well and the OD value was tested by ELIASA after cultured 12, 24, 48, 72, 96 hours. **B-C.** 1000 SiHa cells were planted in the culture plate. The number of colonies presented is the mean of colony counts from 3 different experiments.

Supplementary Table S1: The statistical result of CLDN1 for immunochemistry

Scoring for CLDN1 immunostaining	1+	2+	3+
Normal (n=30)	30(100%)		
CIN I(n=15)	8(53%)	7(47%)	
CIN II(n=15)	6(40%)	6(40%)	3(20%)
CIN III(n=15)	4(27%)	6(40%)	5(33%)
SCC without lymph node metastasis (n=73)	10(14%)	28(38%)	35(48%)
SCC with lymph node metastasis (n=50)	3(6%)	12(24%)	35(70%)
